# Successful Vaginal Delivery of Naturally Conceived Dicavitary Twin in Didelphys Uterus: A Rare Reported Case

**DOI:** 10.1155/2017/7279548

**Published:** 2017-08-27

**Authors:** Houda Nasser Al Yaqoubi, Nishat Fatema

**Affiliations:** Department of Obstetrics and Gynaecology, Ibri Regional Hospital, Ministry of Health, Ibri, Oman

## Abstract

Didelphys uterus, or double uterus, is an embryological developmental malformation of the müllerian ducts with the incidence of approximately 8.3% of all müllerian duct abnormalities (MDAs). Didelphys uterus accompanying dicavitary twin gestation is encountered as a very rare entity with overall incidence of about 1 in 1,000,000. We report a rare case of didelphys uterus, diagnosed since her first pregnancy, and during her fourth pregnancy she conceived dicavitary twin naturally without any infertility treatment. Though, the pregnancy course was complicated by preterm labour at 34-week gestation and she delivered simultaneously both fetuses with the cephalic presentation by spontaneous vaginal delivery with good maternal and neonatal outcomes.

## 1. Introduction

Didelphys uterus is an embryological developmental malformation of the müllerian or Wolffian ducts, characterized by complete failure of the müllerian ducts to fuse, resulting in two separate uterine cavities and cervices. Sometimes a longitudinal or transverse vaginal septum varying from thin and easily displaceable to thick and inelastic may also be associated with didelphys uterus [1, 2].

Among müllerian duct anomalies (MDAs), the septate uterus is the most common (35%) followed by bicornuate uterus (25%), arcuate uterus (20%), then unicornuate (9.6%), and complete agenesis (3%). The occurrence of didelphys uterus is the second least common with the incidence of approximately 8.3% of all MDAs. The prevalence of didelphys uterus is reported to be 1 in 1000–1 in 30,000 women [1].

These uterine anomalies are associated with delayed natural conception and subfertility. In case of infertile women, uterus didelphys has been found around 0.2%. For such cases, successful pregnancies can be achieved by artificial reproductive technique and embryo transfer [2, 3].

Natural twin conception in each cavity of didelphys uterus is a very rare entity and only a handful of cases are reported in the literature to date. Because of its rarity, didelphic uterus accompanying natural dicavitary twin conception without any fertility treatment has not been researched extensively in the literature, so the exact rate of occurrence is unknown in general population [1, 4].

Dicavitary twin in a double uterus was first described in 1927. The overall incidence of dicavitary twin gestation (ART/Natural conception) in uterus didelphys has been reported approximately 1 in 1,000,000 [1, 3].

Like other MDAs, uterine didelphys is associated with various obstetric complications like spontaneous miscarriages, malpresentation, preterm delivery, preterm rupture of membrane, intrauterine growth restriction, and the need for operative delivery [4, 5].

We are discussing an extremely rare case of a naturally conceived dicavitary twin pregnancy in didelphys uterus, who had a successful vaginal delivery of both fetuses simultaneously without any complications at 34 weeks of gestation.

## 2. Case Presentation

A 30-year-old G4P1Ab2 woman was diagnosed by MRI to have uterus didelphys with two cervices and longitudinal upper vaginal septum since her first pregnancy. During her fourth pregnancy, she was booked with us at 10 weeks of gestation with a singleton fetus in each cavity (dicavitary twin) of the uterus didelphys.

Her past obstetric history revealed that she had two first trimester miscarriages followed by IUI (intrauterine insemination) conception in her third pregnancy. In her third pregnancy, induction of labour (IOL) was done at 36 weeks of gestation in view of intrauterine growth restriction (IUGR). Following IOL, she delivered vaginally an alive preterm baby with the weight of 2 kg. Her medical history is significant for hypothyroidism and she is on Tab. Thyroxin 25 microgram daily.

**Figure 1:**
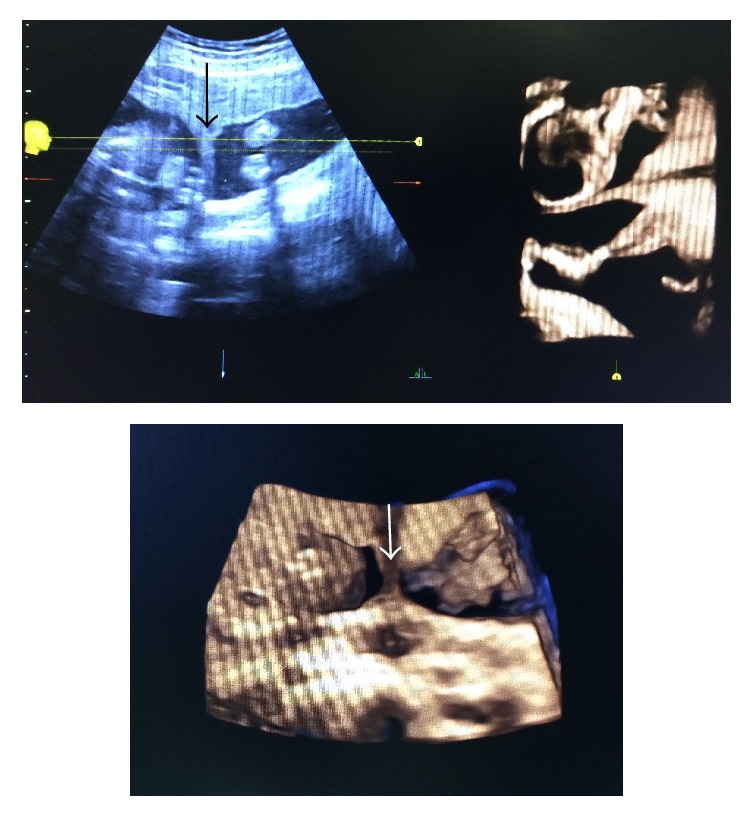
Thick septum separating the uterine cavities showing in 2D and 3D ultrasound mode. The arrows showed thick septum separating the uterine cavities showing in 2D and 3d ultrasound mode.

During her current pregnancy, she conceived naturally without any fertility treatment. After booking, she was referred for routine antenatal care at Maternal-Fetal Medicine (MFM) clinic due to twin gestation and uterine anomaly and for the risks associated with it. On subsequent follow-up in MFM clinic, she was diagnosed as dicavitary twin pregnancy in didelphys uterus by USG (Figure 1). Her blood group was AB +ve and booking hemoglobin was 11.7 gm/dl.

She was started on low dose aspirin (75 mg) in view of the previous history of IUGR baby. She followed up biweekly at the MFM clinic. Detailed anatomical analysis of both fetuses was done by ultrasound during second trimester. The analysis revealed normal amniotic fluid and anteriorly placed placenta for both fetuses. The length of both cervices was within normal limit.

During 33 + 3 weeks of gestation at the time of her routine antenatal follow-up, she complained of intermittent premature contraction, so vaginal examination was performed which revealed left cervix 1.5 cm long os 2 cm dilated membrane intact and station at −3 and the other cervix was closed.

She was admitted for observation; two doses of dexamethasone (12 mg) were given 12 hours apart for fetal lung maturation. Fetal growth for both fetuses by USG: first fetus (right uterus) was with estimated weight 1.7 kg and normal amniotic fluid, and the second fetus (left uterus) estimated weight was 1.9 kg and normal amniotic fluid. Umbilical artery Doppler for both fetuses was normal (Figure 3). Cardiotocograph tracing for both fetuses was satisfactory. Two days later she was discharged and plan of delivery was discussed with the couple in detail; if both babies remained cephalic and no complications arise, she will be allowed for the trial of vaginal delivery. If there was malpresentation or any element of fetal distress of any one of the fetuses, then the cesarean section would be considered for both fetuses. She and her husband agreed with our plan.

One week after discharge from the hospital, at 34 + 3 weeks of gestation, she was presented with preterm labour. On physical examination, she was vitally stable and was getting the strong uterine contraction and vaginal examination revealed that she was in the second stage of labour. Immediately after admission, the first twin was delivered and after delivery of first twin amniotomy was done for the second twin. Around 11 minutes after the delivery of first twin, the second twin was delivered from the other uterine cavity. Both placentas were removed smoothly from the separate uterine cavities. The neonatal outcomes were good with Apgar scores for both babies of 9 in 1 minute and 10 in 5 minutes. The first baby was a male with weight 1.6 kg, and the second baby was female with weight 2 kg. Neonates were kept in the neonatal intensive care unit for preterm care and observation. Neither the patient nor the neonates have experienced any other complications. Patient's postnatal course was uneventful and on the second postnatal day she was discharged in good condition with her healthy babies.

## 3. Discussion

We presented a case of known uterus didelphys with naturally conceived dicavitary twin pregnancy which is an extremely rare occurrence.

**Figure 2:**
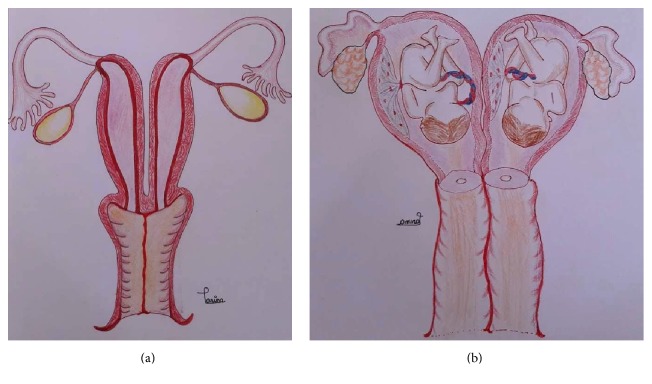
(a) Schematic diagram of nongravid didelphys uterus and (b) schematic diagram of gravid didelphys uterus with dicavitary twin.

**Figure 3:**
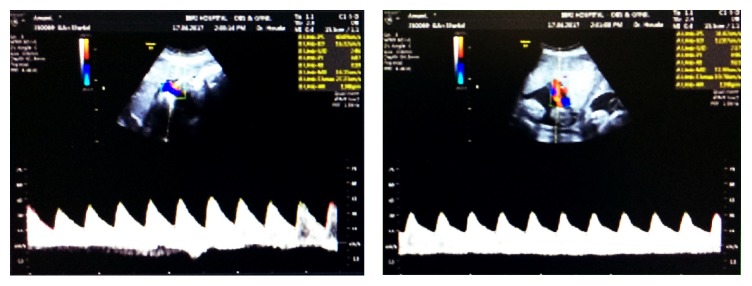
Umbilical artery Doppler for both fetuses at 33 weeks of gestation.

The failure of fusion of the müllerian ducts results in uterus didelphys. It is a developmental abnormality of müllerian ducts comprised the double uterus with completely developed independent horns including endometrium, myometrium, and serosal layers; two cervices; and longitudinal or transverse vaginal septum (Figure 2(a)). The etiology of uterus didelphys is not known exactly with the frequency from 1 in 1000 to 1 in 30,000 women [4, 5]. Didelphys uterus accompanying naturally conceived twin pregnancy with each fetus in the separate cavity (Figure 2(b)) is a rare entity. Only a few number of cases have been reported, but no large series exist in the literature [1, 6].

To the best of our knowledge, not more than 20 cases of dicavitary twin or multiple gestation in didelphys uterus have been researched to date [6, 7].

The exact incidence of the condition is unknown. The overall incidence of dicavitary twin gestation conceived either spontaneous or by the artificial reproductive technique is estimated approximately 1 in 1,000,000 [1, 6]. It is hypothesized that these twins are biovular in all cases, where the two ova might come from the two follicles of the same ovary or in both ovaries ovulation may occur during the same cycle [8].

Our patient was diagnosed to have didelphys uterus since her first pregnancy and conceived dicavitary twin, naturally without any infertility treatment. Unfortunately, the pregnancy course was complicated by preterm labour at 34 weeks of gestation and she delivered simultaneously both fetuses with the cephalic presentation by spontaneous vaginal delivery. The time interval between deliveries of both fetuses was only 11 minutes.

Similar to our case, Allegrezza reported a case of natural dicavitary twin pregnancy in didelphys uterus, in which the patient had premature rupture of membrane followed by preterm labour at 31 weeks of gestation, both fetuses were cephalic and delivered vaginally without any complications [4].

The contractions of both uteri may not begin simultaneously. There are reported cases where the delivery interval between the twins varies from several hours or even several weeks [8].

One case is reported by Nohara et al. in which one twin was delivered by cesarean section at 25 weeks of gestation due to fetal distress followed by premature rupture of membrane and another one was delivered vaginally with a 66 days' interval at 35 weeks of gestation. They described that in didelphys uterus as the uterine horns are individually functioning so the initiation of labour could be local rather than systemic control [7].

Maki et al. described another case of dicavitary twins in didelphys uterus that were conceived after fertility treatment, where at 37 weeks of gestation the woman had preterm premature rupture of the membrane of right horn of uterus followed by progression of labour with simultaneous contractions of both horns of the uterus. The fetus in the right horn was delivered by spontaneous vaginal delivery and the second twin was delivered by cesarean section in view of abnormal cardiotocograph (CTG). They analyzed the synchronized contractions of both horns of the didelphys uterus and commented that the primary uterine contractions are caused by the individual rhythms of the bilateral pacemaker sites surrounding the uterotubal junction and subsequently the help of the gap junctions in between both uterine sides resulted in synchronized uterine contractions to expel the uterine contents [6].

In our case, luckily the CTG tracing of both fetuses was reactive, and both were delivered vaginally without any difficulty within an 11-minute time interval.

Only a few cases of twin gestation with didelphys uterus that had spontaneous vaginal delivery are mentioned in the literature [1, 8].

Didelphys uterus is associated with varieties of obstetric complications including early and late miscarriages, malpresentation, intrauterine growth restriction, preterm delivery, and preterm rupture of membrane [4, 5, 9].

Cervical incompetence is not commonly occurred with didelphys uterus so cervical cerclage is not routinely recommended unless there is an evidence of cervical incompetence or dilation either by clinical examination or ultrasonography during early second trimester. A case of didelphys uterus with dicavitary twin was reported with the short cervix at 30 weeks of gestation with uterine contractions. They managed the case by tocolytic therapy with nifedipine until 34 weeks and then at 37 weeks of gestation cesarean section was done for both fetuses due to fetal distress of one twin. They did not observe any adverse effects of tocolytic therapy [1, 2].

The overall obstetric outcome of uterus didelphys is poor but still better than the other MDAs like the septate or bicornuate uterus. The reason behind this occurs is that in didelphys uterus the blood supply through the collateral circulation in between two horns is better in comparison to other MDAs. The successful pregnancy rate with didelphys uterus is 57%, and the fetal survival rate is documented around 64% [2, 4, 10].

In these cases, no specific route of termination of pregnancy is recommended in the literature, though both vaginal and cesarean delivery have been mentioned in the previous studies. The incidence of cesarean section is documented about 82%. If both fetal presentations are cephalic and there are no other associated risk factors, then vaginal delivery can be considered as the mode of delivery [2, 5, 10].

If the cesarean section is indicated then a low midline longitudinal incision is preferable for proper exposure of both uterine cavity to facilitate the delivery of the fetuses [3].

## 4. Conclusion

Most of the previous studies regarding didelphys uterus with twin gestation had the history of fertility treatment, and the termination of pregnancy was required by cesarean section either due to fetal malpresentation or fetal distress. Our presented case is the dicavitary twin with didelphys uterus, which was conceived naturally, and although the pregnancy was complicated by preterm labour, both fetuses were delivered vaginally with good maternal and neonatal outcomes.

Didelphys uterus is associated with a twin pregnancy is a high-risk pregnancy. The early detection of this anomaly of the uterus and accompanying pregnancy by ultrasonography is of great value. Close monitoring of fetal growth, biophysical profile, and the cervical condition is recommended throughout the pregnancy. The time and mode of delivery should be planned and discussed in detail with the couple during antenatal follow-up [4, 5, 8].
